# Diabetic Macular Edema in the Western Part of Romania: Screening to Improve Patient Outcomes

**DOI:** 10.3390/jpm15030106

**Published:** 2025-03-09

**Authors:** Adriana Ivanescu, Simona Popescu, Deiana Roman, Monica Dragomir, Romulus Timar

**Affiliations:** 1 Second Department of Internal Medicine, “Victor Babes” University of Medicine and Pharmacy, 300041 Timisoara, Romania; adriana.ivanescu@umft.ro (A.I.); roman.deiana@umft.ro (D.R.); monica.dragomir@umft.ro (M.D.); timar.romulus@umft.ro (R.T.); 2 Opticlass Ophtalmology Clinic, 300012 Timisoara, Romania; 3 Department of Diabetes, “Pius Brinzeu” Emergency Hospital, 300723 Timisoara, Romania

**Keywords:** diabetes mellitus, diabetic macular edema, health status, comorbidities, multidisciplinary, personalized medicine

## Abstract

**Background**: Diabetes mellitus (DM) is a global healthcare concern with a rising prevalence. Patients with DM have a severely diminished quality of life due to the extensive range of connected complications. One of the most impactful diabetes-associated pathologies is diabetic macular edema (DME), as it is a major cause of blindness globally. Patients with DME present many concomitant diseases that influence their prognosis. The present research seeks to describe the most frequent DME-related comorbidities. **Method**: This study enrolled 105 participants previously diagnosed with type 1 DM (T1DM) or type 2 DM (T2DM) (77 presenting with DME), who were evaluated regarding other associated comorbidities. **Results**: Patients in the DME group presented a median age of 65, with a mean disease duration of 15 years and inadequate glycemic control, reflected by a mean HbA1c of 7.5%. All patients presented at least one comorbidity, with hypertension (100%) and dyslipidemia (62.3%) being the most prevalent. Spearman analysis revealed a statistically significant correlation between DME and diabetes duration (*p* = 0.01), proliferative diabetic retinopathy (*p* = 0.004), and chronic kidney disease (*p* = 0.034). **Conclusions**: Patients with DME often present multiple comorbidities that must be screened for and addressed through a multidisciplinary approach.

## 1. Introduction

Diabetes mellitus (DM) is a chronic metabolic disease characterized by continuous hyperglycemia. According to the International Diabetes Federation’s (IDF) 10th edition, the worldwide prevalence of diabetes is rising at an alarming rate, posing a significant challenge to public health [1]. DM is a leading contributor to major health issues, including cardiovascular diseases (CVD) such as acute myocardial infarction and strokes, as well as other severe complications like blindness and chronic kidney disease (CKD) [2].

Diabetic eye disease comprises several pathological changes, which include, among others, diabetic retinopathy (DR), diabetic macular edema (DME), glaucoma, cataracts, and even changes involving the ocular surface [3].

DME, with a continuously rising prevalence, is one of the most serious complications related to DM, as it is one of the leading causes of vision impairment worldwide [4]. DME can occur in any stage of DR, and its presence requires prompt and decisive therapeutic intervention in order to prevent progression and irreversible retinal damage while simultaneously lowering the risk of blindness in patients with DM [5].

DME is characterized by retinal thickening, mostly in the outer and inner plexiform layers, mainly determined by a breakdown of the blood–retinal barrier [6]. This abnormal accumulation of fluid, lipids, and proteins involves the macular area and frequently impacts the fovea, directly leading to decreased visual acuity among patients with DM [2].

DME prevalence varies significantly, ranging from 4.2% to 14.3% in individuals with T1DM and from 1.4% to 5.57% in those with T2DM [7,8]. The high prevalence of DME not only severely affects patients’ quality of life but also places a heavy economic overload on healthcare systems worldwide [2].

DME has a multifactorial nature, with a wide range of factors involved in its development. Gender, ethnicity, diabetes type/duration, or genetic factors represent unmodifiable factors, while hypertension (HTN), dyslipidemia, obesity, and chronic kidney disease are the most frequent pathologies linked to DME. Last but not least, habits like smoking or alcohol consumption also have a negative impact on DME evolution [9].

It is well known that patients with DM in general, not only those with DME, exhibit a higher risk of associated health problems than those who do not have DM. Approximately 60–70% of individuals with diabetes have at least one comorbidity, while 25–30% already present with more than two comorbidities at the time of their DM diagnosis. However, in everyday clinical practice, when these patients are screened for diabetes-related ocular complications, overall health status might be somewhat disregarded, resulting in overlooking potential factors that impact the development of these complications, especially DME. Many patients with DM, even those recently diagnosed, may already present chronic complications due to the prolonged metabolic dysregulation that often precedes the formal diagnosis of DM. For example, DM has been associated with changes in intraocular pressure (IOP). A recent study found that diabetes progression and altered glycemic control are directly linked to increased IOP before resulting in glaucoma [10]. Therefore, a thorough general and ophthalmologic evaluation is mandatory in all diabetic patients [11].

Regarding the systemic management of DME and other diabetes-related complications, the general consensus among healthcare professionals still involves glycemic control. However, considering the complex health status of patients with DM and the multiple risk factors involved in DME development, additional measures must be taken, such as strict blood pressure control, lowering lipid levels, and the proper management of obesity and renal status [12]. Last but not least, smoking and alcohol consumption cessation must be recommended in all patients with DM, despite the degree of retinal complications. These suggest that improving outcomes for patients with DME may require a broader approach, incorporating the investigation of additional risk factors and innovative treatment strategies beyond glycemic management alone [13].

The aim of this research was to evaluate the general health status of patients with DME, focusing on the diseases most frequently related to both DME and DM, in general, in order to raise awareness regarding the need for more stringent healthcare management of these patients, while also observing which are the most important risk factors implied in the appearance and evolution of DME, in order to more promptly screen and take preventative measures to ensure the prevention of blindness.

## 2. Materials and Methods

### 2.1. Study Design and Patient Selection

In this retrospective cross-sectional study, we included patients previously diagnosed with either T1DM or T2DM. All the patients took part in their prescheduled appointments at the Diabetes Care Center within the “Pius Brinzeu” Emergency County Hospital in Timisoara, Romania.

We examined the medical records of 227 patients from the Diabetes Center between 16 July 2024 and 6 August 2024, and we enrolled a total number of 105 patients (55 male and 50 female subjects). 122 patients declined to participate in this research or did not comply with the study’s eligibility criteria. The inclusion criteria comprised patients above 18 years of age with a prior DM diagnosis (either T1DM or T2DM). Exclusion criteria included other DM subtypes (gestational and secondary DM), significant cognitive impairment or other neuro-psychiatric disorders that prevented patients from giving informed consent, comorbidities that necessitated hospitalization during our study, institutionalized patients, and patients who presented any ocular pathologies that would interfere with a proper retinal examination (advanced cataracts and significant vitreous hemorrhage). Other retinal diseases that could interfere with an adequate classification of DME and DR by altering the examined retinal structures (severe myopia, detachment of the retina, or significant macular pucker) were also excluded from this research.

The protocol for the present study was approved by the Ethics Committee of the “Pius Brinzeu” Emergency County Hospital, Timisoara, Romania (approval no. 472/15.07.2024), adhering to the principles outlined in the Declaration of Helsinki, revised in 2013, and its subsequent amendments.

### 2.2. Data Collection and Medical Assessment

The following patient data were retrieved: gender, age, weight and height, diabetes type, disease duration, current medication, comorbidities, and smoking status. Additionally, a complete fundus examination was performed by a trained ophthalmologist, using a Topcon SL-D2 biomicroscope.

The assessment of biological parameters comprised a complete blood panel for all patients during their visit at the Diabetes Care Center. Diagnosis of DM was previously established through the presence of a fasting plasma glucose level > 7.0 mmol/L (126 mg/dL), 2 h post-load plasma glucose > 11.1 mmol/L (200 mg/dL), or HbA1c level above 6.5% (48 mmol/mol). Disease duration refers to the number of years from the initial DM diagnosis to the date of inclusion in the present research. Weight and height were measured for all participants, and the Body Mass Index (BMI) was calculated using the metric system. Following the World Health Organization (WHO) criteria regarding obesity, patients with BMI values above 25 kg/m^2^ were considered overweight, while those with BMI equal to or above 30 kg/m^2^ were diagnosed with different degrees of obesity. Blood pressure was also evaluated in all patients with the aid of an aneroid sphygmomanometer and hypertension was diagnosed in the presence of a systolic blood pressure value above or equal to 140 mmHg and a diastolic blood pressure above or equal to 90 mmHg. Meanwhile, CVD presence was derived from patients’ medical history and referred to either a previous diagnosis of coronary heart disease or cerebrovascular disease. Diabetic neuropathy was evaluated by performing nerve conduction velocity (NCV), with values less than 40 m/s being considered pathological, as well as with the usage of the Michigan Neuropathy Screening Instrument (MNSI) with resulting clinical scores above 2.5, questionnaire scores above 7, or overall scores over 9.5. Chronic kidney disease (CKD) was determined at values below 60 mL/min of the creatinine-based glomerular filtration rate (eGFR) estimate. Dyslipidemia was diagnosed based on lipid panel values. Smoking habits were self-reported.

A board-certified ophthalmologist performed an extensive fundus examination after prior pupil dilation on all patients with a Topcon SL-D2 biomicroscope (Tokyo, Japan). Cataract diagnosis or prior cataract surgery with intraocular lens implantation (IOL) was reported. Retinal structures were examined and DME and DR diagnosis were established according to the Early Treatment Diabetic Retinopathy Study Design (ETDRS) guidelines [14]. DME was considered in the following circumstances: retinal thickening or the presence of hard exudates within 1 disk diameter from the center of the fovea [15].The presence of microaneurysms, hemorrhages, exudates, venous beading, or neovascularization was taken into account in DR staging as follows: non-proliferative diabetic retinopathy (NPDR) with mild, moderate, and severe NPDR stages and proliferative diabetic retinopathy (PDR) [16]. IOP measurement was performed for all patients with a Perkins handheld applanation tonometer (Perkins Mk3 tonometer, Haag-Streit, Koniz, Switzerland). Patients’ medical charts were also reported prior to the ophthalmologic treatments, including retinal laser photocoagulation or intravitreal injections with anti-VEGF.

### 2.3. Statistical Analysis

Statistical analysis was performed using the IBM Statistical Package for Social Sciences software (version 28, Armonk, NY, USA, accessed on 1 August 2024, https://www.ibm.com). The characteristics of the two study subgroups were presented using descriptive statistics, including the median, percentage, and interquartile range (IQR), depending on the variable type. The normality of the data distribution was evaluated using visual tools, such as histograms and probability plots, as well as analytical tests, specifically the Shapiro–Wilk test. Since the data were not normally distributed, numerical variables were reported as medians with the 25th and 75th IQRs, and the Mann–Whitney U test was used for comparisons between groups. Categorical variables were described as frequencies and percentages, with comparisons among groups performed using the Chi-squared or Fisher’s exact test, depending on the data. The relationship between DME and the risk factors T1DM and T2DM was assessed using the point-biserial correlation (rpb) for continuous variables and the Phi coefficient (ϕ) for categorical variables. We also conducted a multivariate logistic regression to determine potential risk factors for DME within the study population. A *p*-value (two-tailed) of less than 0.05 was considered statistically significant.

## 3. Results

### 3.1. General Characteristics of the Study Population

The study included 105 participants, divided into two groups based on DME presence (*n* = 77) or absence (*n* = 28). The median age of the participants was 66 (IQR: 59–73) years. Those within the DME group were observed to be younger; however, this difference did not present a statistical significance value (*p* = 0.071). Both groups were similar regarding gender distribution, as seen in Table 1.

**Figure 1:**
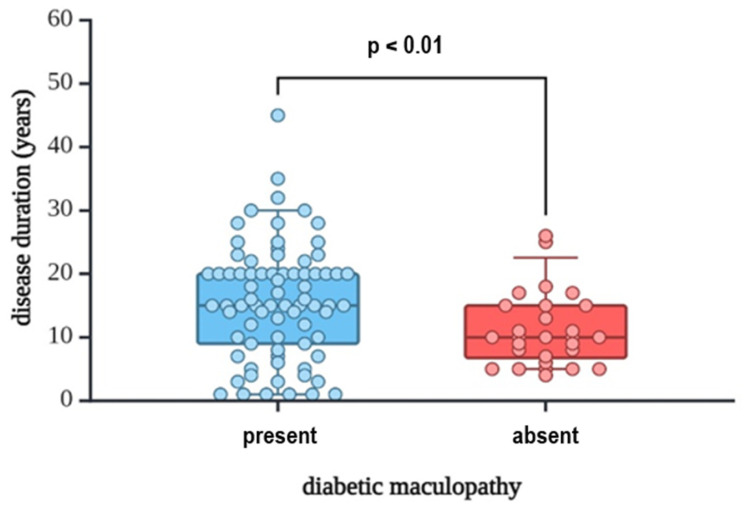
Disease duration and diabetic maculopathy status. Circles represent individual data points. Created with Biorender.com.

A total of 92.4% of the total study population had T2DM, and the rest T1DM. Disease duration was significantly longer in the DME group (median 15 years) compared to the group without DME (median 10 years), as seen in Figure 1. This indicates that patients with diabetes and DME had a longer DM duration than those without DME.

In terms of diabetes management, most of the patients were treated with non-insulin antidiabetic drugs, 30.5% were under treatment with insulin, and 12.4% received a combination of the two. There was no statistically significant difference in the distribution of these treatments between groups (*p* = 0.219). The current and maximum HbA1c levels were recorded, with the median values were slightly higher in the DME group (current HbA1c: 7.50%, IQR: 6.72–8.27; maximum HbA1c: 10.90%, IQR: 8.95–13) compared to the group without DME (current HbA1c: 6.85%, IQR: 6.42–7.80; maximum HbA1c: 9.45%, IQR: 8.85–10.70). The prevalence of dyslipidemia, HTN, obesity, and CVD comorbidities did not differ notably between the two studied groups. CKD was present only among patients who also presented with DME. Diabetic neuropathy was also more common in patients with an associated diagnosis of DME, though this finding did not reach statistical significance. Still, all patients with DME had HTN in the researched population, 48.1% of them presenting moderate HTN, while dyslipidemia was diagnosed in 62.3% of the same subgroup. Furthermore, almost half of this subgroup presented with at least one other comorbidity (46.8%).

### 3.2. Ophthalmic Findings

Intraocular pressure (IOP) measurements in both the right eye (RE) and left eye (LE) did not vary significantly between groups. DR presence was significantly associated with DME (*p* < 0.001), with a greater proportion of patients in the DME group having PDR, as seen in Table 2. This led to the conclusion that macular edema was not only associated with diabetic retinopathy but also with more severe cases of the disease.

The use of intravitreal injections and laser photocoagulation was significantly more common in the DME group (both *p* < 0.001), indicating the need for more aggressive ophthalmologic intervention in these patients.

### 3.3. Correlation Analysis of DME with Risk Factors for T1DM and T2DM

Correlation analysis was conducted to assess which demographic or clinical factors correlated with the presence of DME (Table 3). A strong positive correlation was detected with DR (*r_pb_* = 0.643), while weaker correlations were found with disease duration (*r_pb_* = 0.246), current HbA1c (*ϕ* = 0.241), and CKD (*r_pb_* = 0.206).

### 3.4. Association Between DME and Several Demographic and Clinical Factors

In order to obtain more information regarding DME in patients with DM, a multivariate logistic regression analysis was performed, investigating potential factors associated with DME, as shown in Table 4. After adjusting for multiple confounding factors, including risk factors T1DM and T2DM, only PDR remained statistically significant as an independent factor linked to DME occurrence (*p* = 0.004).

## 4. Discussion

The global prevalence of DME is increasing. As the number of DM cases rises, a corresponding rise in complications related to diabetes is expected [17]. With the worldwide increase in the aging population [18], the concept of multimorbidity has received significant attention among medical practitioners [19], particularly regarding patients with T2DM, who present a greater risk of having numerous co-occurring conditions [20]. Our research focused on observing DME cases in the western Romanian population with T2DM, which is known to be highly prevalent in this part of the country. Conditions such as arterial hypertension, chronic kidney disease, and obesity have a major impact on ocular complications related to DM, including vision-threatening DME [21].

In our study group, those who suffered from DME had longer DM duration, with a median of 15 years (*p* = 0.010), data that align with the current literature findings, which report a direct correlation between diabetes duration and the development and progression of ophthalmologic complications such as DME and DR. Notably, patients with T2DM might present DME at the time of DM diagnosis, underlining the importance of screening in both recently diagnosed patients, as well as patients who have been diagnosed for a longer period of time [6,22]. Regarding glycemic control, HbA1c levels were suboptimal in the whole research group but were particularly elevated among patients with DME (median value 7.5%, maximum value 10.9%). Increased HbA1c values are known to be linked to DME occurrence and progression, while conversely, adequate glycemic control positively impacts ocular-related complications. According to DDCT/EDIC, a 10–25% decrease in the risk of DME occurrence can be obtained by only a 1% decrease in HbA1c levels [23].

DR severity is the major risk factor linked to DME. Although DME can be found at any degree of DR, an increase in DR severity has been associated with the rising prevalence of DME [14]. Our research found a strong positive correlation between the presence of DR and DME (*p* = 0.643). Moreover, PDR was a statistically significant factor involved in DME occurrence (*p* = 0.004). Intravitreal injections and retinal laser photocoagulation prevailed as treatment strategies among our population, emphasizing the need for a more decisive approach when facing diabetes-related ocular complications. This situation seriously impacts DM patients’ quality of life [24]. Even so, treatment outcomes do not always meet the desired results, suggesting other general health factors may have a greater impact on visual outcomes [25].

More than 60% of patients with DM present at least one comorbidity at the time of their DM diagnosis [26]. Our research population aligns with these findings, as the majority of the evaluated patients presented at least one comorbidity, arterial hypertension and dyslipidemia being the most prevalent pathologies among our DME subgroup. All these components lead to the appearance of metabolic syndrome, which in turn increases the risk of cardiovascular complications, one of the most serious ones being atrial fibrillation. Also, DME and metabolic syndrome are closely linked through their shared pathophysiological mechanisms, primarily related to insulin resistance, chronic inflammation, and vascular dysfunction [27]. All DME patients were diagnosed with HTN, with different degrees of severity, presenting a slight predominance towards moderate HTN (48.1%), according to data sustained by a study conducted by Zhang M et al. [28]. On the other hand, HTN presence, regardless of the severity, is linked to an increase in the probability of DME occurrence of 40% [29]. The Wisconsin Epidemiologic Study of Diabetic Retinopathy found that systemic hypertension triples the prevalence of DME. It has been proposed that hypertension serves as a risk factor for the development of macular edema, and its management could provide significant benefits for patients with uncontrolled blood pressure. Sustaining the importance of HTN management, according to UKPDS 69, a reduction of only 10 mmHg in systolic blood pressure reduced the risk of DME development by 15% [30], while the optimal blood pressure values assessment among patients with DM could be represented by a 24 h measurement, due to the more significantly predictive power concerning the evaluation cardiovascular risk in these patients [31].

Previous studies have indicated that dyslipidemia is linked to DME. Furthermore, a global multicenter study found that high triglyceride levels and low HDL cholesterol levels were associated with an increased risk of diabetic microvascular diseases. Dyslipidemia is one of the most frequent comorbidities found in patients with DM [26]. Our research revealed that 66.7% of evaluated patients presented this pathology, 62.3% belonging to the DME subgroup. Treating dyslipidemia should address all cholesterol fractions, yet elevating HDL-cholesterol levels must become a therapeutic target, especially among patients with DME, as it was found to decrease the risk of DME, as stated by Klein et al. and confirmed by another study conducted by Chung et al. [32,33].

Besides HTN and dyslipidemia, 46.8% of the DME subpopulation presented at least one other comorbidity, such as CKD, diabetic polyneuropathy, or CVD. Regarding these comorbidities, we found a statistically significant correlation between CKD and DME (*p* = 0.034). Multiple reports have evaluated the relationship between DME and CKD. Acan et al. reported CKD is a risk factor for DME development [13], while another study from Melbourne observed no associations between GFR and DME in 61 Caucasian patients with DME [34]. In contrast, another population-based study indicated that CKD was significantly associated with incident DME during a 10-year follow-up examination [35]. The positive association between DME and GFR was also sustained by a single-center retrospective observational study involving patients with T2DM in South China [36]. These different results among various studies suggest that although both CKD and DME share a comparable pathology mechanism, microangiopathy, the exact influence of renal factors on DME remains unclear [37].

Lifestyle-related habits are also of great importance when deciding upon a therapeutic approach for a patient with DM and DME [38]. Obesity is known to be especially prevalent in patients with T2DM, and its management becomes mandatory, as lowering body mass is known to have a beneficial effect on visual outcomes post-DME treatment [39]. In our research, 27.3% of DME patients had obesity, results that the relatively small research group might determine. We consider that further research is necessary in this direction, as there are studies that link not only BMI values but also abdominal fat distribution (waist-to-hip ratio) to the development of diabetes-related ocular pathologies [39,40].

Regarding smoking addiction, we have found that 24.7% of patients with DME were active smokers. Since smoking was self-reported, we did not take past or occasional smokers into account, as we considered it would impede result precision. However, we underscore the need for these categories to be included in future research. Data regarding the direct impact of smoking on DME is somewhat inconclusive in the existing literature. Still, smoking cessation should be advised in all patients with DM, as nicotine is known to impair insulin activity and negatively impact glycemic control [41,42].

As shown in existing studies and as we have observed in the present study, most of the patients with DME have multiple associated diseases that facilitate the appearance of macular edema and accelerate its progression. For this reason, it is very important to identify these pathologies as early as possible so that through their correct management, we can try to prevent the occurrence and subsequent progression of DME.

Although somewhat restricted by the relatively small population size, this research accurately reflects the general health status among patients with DM with DME evaluated in hospital settings in the western part of our country, and the baseline health characteristics of the studied cohort are reflected with precision. Furthermore, considering the consecutive patient enrollment in this research, the heterogeneity of the final study group emphasizes the diversity of patients that medical practitioners encounter in daily clinical practice, accentuating the need for a personalized medical approach for each patient.

Our results have shown that patients with DME generally present multiple associated comorbidities. Therefore, a stringent treatment plan is mandatory, which is an objective achieved in Romania for DM patients by regular check-ups every 3 months, while chronic complication screening is usually performed annually. These particular visits also address patient education regarding diabetes and diabetes treatment. Yet, further education is needed concerning the associated general and ocular diseases and their symptoms to better understand the disease and facilitate better treatment adherence among patients. Besides an increase in the degree of health education, as multiple comorbidities threaten each diabetic patient’s quality of life, we consider that general and ophthalmologic health status screening should be performed at least every 6 months, as a proper diagnosis timing could lead to the more personalized and effective management of these patients.

Regarding the ophthalmologic evaluation of diabetic patients, while medical settings equipped with optical coherence tomography (OCT) would represent the ideal scenario since it represents the gold standard in the diagnosis of DME and other diabetes-related pathologies, the patients often do not have access to hospitals equipped as such. As such, it is important that screening is performed for all patients with DM, whether at the initial consultation or well into their diagnosis [43]. Still, we emphasize the importance of an extended ophthalmologic examination using fundus photography and OCT for a personalized treatment protocol and a precise follow-up.

Future research perspectives also refer to expanding the study population to a larger and younger demographic group while conducting a multicenter, prospective study for a more accurate representation of diabetes-related ocular complications such as DME and associated comorbidities and their interrelation, the main objective remaining individualized patient management and the subsequent quality of life improvement.

## 5. Conclusions

This body of research underlines the importance of close monitoring for DME in patients with DM, underlining the risks represented by this complication and the major impact on the quality of life of these patients while simultaneously identifying individuals for which DME is more prevalent. In all evaluated patients associated with at least one other comorbidity, HTN, dyslipidemia, or CKD were more prevalent and are known to be the most significant risk factors involved in DME occurrence.

Considering the continuously growing numbers of people with DM worldwide, the necessity of personalized medicine becomes more and more evident, especially given the multitude of complications that accompany this disease. A personalized approach could translate into early complication detection, better quality of life, and improved morbidity and mortality rates. In this particular case, patients with DM would benefit from early DME detection and subsequent management, guaranteeing sight preservation.

## Figures and Tables

**Table 1:** General characteristics of the study population.

Variables	Total (*n* = 105)	DME (−) (*n* = 28)	DME (+) (*n* = 77)	*p*-Value
Age (years)	66 (59, 73)	69.5 (60.2, 74)	65 (58, 71.5)	0.071
Male *n* (%)	55 (52.4)	14 (50)	41 (53.2)	0.768
T1DM/T2DM *n* (%)	8 (7.6)/97 (92.4)	1 (3.6)/27 (96.4)	7 (9.1)/70 (90.9)	0.679
Disease duration	15 (8, 20)	10 (6.25, 15)	15 (9, 20)	**0.010**
Diabetes treatment *n* (%)				0.219
• OAD	58 (55.2)	19 (67.9)	39 (50.6)	
• INS	32 (30.5)	7 (25)	25 (32.5)	
• OAD + INS	13 (12.4)	1 (3.6)	12 (15.6)	
• OAD + weekly injection	2 (1.9)	1 (3.6)	1 (1.3)	
Current HbA1c %	7.30 (6.50, 8.07)	6.85 (6.42, 7.80)	7.50 (6.72, 8.27)	0.098
Maximum HbA1c %	10.1 (8.95, 12.55)	9.45 (8.85, 10.70)	10.90 (8.95, 13)	0.102
Dyslipidemia *n* (%)	70 (66.7)	22 (78.6)	48 (62.3)	0.161
HTN *n* (%)				0.292
• Mild	29.5 (31)	7 (25)	24 (31.2)	
• Moderate	48 (45.7)	11 (39.3)	37 (48.1)	
• Severe	26 (24.8)	10 (35.7)	16 (20.8)	
Obesity *n* (%)	31 (29.5)	10 (35.7)	21 (27.3)	0.402
Comorbidities *n* (%)	42 (40)	15 (53.6)	27 (35.1)	0.087
• CKD	11 (10.5)	0 (0)	11 (14.3)	**0.034**
• Diabetic neuropathy	13 (12.4)	1 (3.6)	12 (15.6)	0.177
• CVD	18 (17.1)	5 (17.9)	13 (16.9)	0.907
Smoking *n* (%)	22 (21)	3 (10.7)	19 (24.7)	0.120

Mann–Whitney U test and either the Chi-square (χ^2^) test, Fisher’s exact test, or Bowker’s test, as appropriate. Data are expressed as the median, interquartile range (IQR), or percentage (*n*, %). DME, diabetic macular edema; DM, diabetes mellitus; OAD, oral antidiabetics; INS, insulin; HbA1c, hemoglobin A1c; HTN, hypertension; CKD, chronic kidney disease; CVD, cardiovascular disease. Differences that are statistically significant, defined as those with a value of *p* < 0.05, are highlighted in bold.

**Table 2:** Ophthalmologic characteristics of the study population.

Variables	Total (*n* = 105)	DME (−) (*n* = 28)	DME (+) (*n* = 77)	*p*-Value
IOP—RE mmHg	15 (14, 17)	16 (15, 17.7)	15 (13, 17)	0.204
IOP—LE mmHg	16 (14, 18)	16 (15, 18)	16 (13, 18)	0.266
Cataract *n* (%)				
• Absent	15 (14.3)	5 (17.9)	10 (12.9)	0.528
• Present	41 (39)	9 (32.1)	32 (41.6)	0.498
• Pseudophakia	49 (46.7)	14 (50)	35 (45.5)	0.515
Diabetic retinopathy *n* (%)				
• Absent	16 (15.2)	15 (53.5)	0	**<0.001**
• Mild non-proliferative	4 (3.8)	3 (10.7)	2 (2.6)	0.084
• Moderate non-proliferative	9 (8.6)	2 (7.2)	7 (9.1)	1
• Severe non-proliferative	34 (32.4)	6 (21.4)	28 (36.4)	0.166
• Proliferative	42 (40)	2 (7.2)	40 (51.9)	**<0.001**
Intravitreal injections *n* (%)	87 (82.9)	17 (60.7)	70 (90.9)	**<0.001**
Laser photocoagulation *n* (%)	56 (53.3)	6 (21.4)	50 (64.9)	**<0.001**

Mann–Whitney U test and either the Chi-square (χ^2^) test or Fisher’s exact test, as appropriate. Data are expressed as the median (interquartile range, IQR) or percentage (*n*, %). IOP, intraocular pressure; RE, right eye; LE, left eye; DME, diabetic macular edema. Statistically significant differences, with a *p* < 0.05, are highlighted in bold.

**Table 3:** Correlation between DME and risk factors in T1DM and T2DM.

	Age	Gender	Disease Duration	Current HbA1c	Dys-Lipidemia	HTN	CKD	Diabetic Neuropathy	Smoking	DR
*p*-Value	0.070	0.768	**0.011**	**0.014**	0.119	0.117	**0.035**	0.098	0.273	**<0.001**
Correlation coefficient	−0.178	−0.029	0.246	0.241	0.152	−0.153	0.206	0.161	0.107	0.643

Point-biserial correlation (rpb) and Phi coefficient (ϕ), as appropriate. Abbreviations: DME, diabetic macular edema; HbA1c, hemoglobin A1c; HTN, hypertension; CKD, chronic kidney disease; Statistically significant differences, with a *p* < 0.05, are highlighted in bold.

**Table 4:** Risk factors associated with DME in the study population.

Variables	Model 1		Model 2		Model 3	
	OR (95%CIs)	*p*-Value	OR (95%CIs)	*p*-Value	OR (95%CIs)	*p*-Value
Age	0.887 (0.438, 2.260)	0.802	0.922 (0.343, 2.476)	0.871	1.135 (0.388, 3.322)	0.818
Gender	0.948 (0.894, 1.005)	0.702	0.948 (0.890, 1.010)	0.970	0.954 (0.893, 1.020)	0.169
Disease duration	1.069 (1.005, 1.137)	**0.034**	1.088 (1.014, 1.166)	**0.018**	1.066 (0.990, 1.148)	0.089
Current HbA1C	1.312 (0.852, 2.021)	0.218	1.349 (0.843, 2.160)	0.212	1.088 (0.654, 1.809)	0.744
Severe HTN			0.471 (0.164, 1.351)	0.161	0.385 (0.119, 1.248)	0.112
Dyslipidemia			0.353 (0.113, 1.101)	0.073	0.500 (0.150, 1.674)	0.261
PDR					10.715 (2.16, 52.97)	**0.004**

Model 1 adjusted for: age; gender; disease duration; current HbA1c. Model 2 adjusted for: age; gender; disease duration; severe HTN; dyslipidemia. Model 3 adjusted for: age; gender; disease duration; severe HTN; dyslipidemia; PDR. OR, odds ratio; CI, confidence interval; HbA1c, hemoglobin A1c; HTN, hypertension; PDR, proliferative diabetic retinopathy. Differences that are statistically significant, defined as those with a probability value of *p* < 0.05, are highlighted in bold.

## Data Availability

All available data can be provided upon request to the corresponding author.
